# Hyperspectral Imaging Coupled with Multivariate Analysis and Image Processing for Detection and Visualisation of Colour in Cooked Sausages Stuffed in Different Modified Casings

**DOI:** 10.3390/foods9081089

**Published:** 2020-08-10

**Authors:** Chao-Hui Feng, Yoshio Makino, Juan F. García Martín

**Affiliations:** 1 Graduate School of Agricultural and Life Sciences, The University of Tokyo, 1-1-1, Yayoi, Bunkyo-ku, Tokyo 113-8657, Japan; 2 RIKEN Centre for Advanced Photonics, RIKEN, 519-1399 Aramaki-Aoba, Aoba-ku, Sendai 980-0845, Japan; 3 Departamento de Ingeniería Química, Facultad de Química, Universidad de Sevilla, 41012 Seville, Spain; jfgarmar@us.es

**Keywords:** sausages, casings, core colour, discriminant analysis

## Abstract

A hyperspectral imaging system was for the first time exploited to estimate the core colour of sausages stuffed in natural hog casings or in two hog casings treated with solutions containing surfactants and lactic acid in slush salt. Yellowness of sausages stuffed in natural hog casings (control group, 20.26 ± 4.81) was significantly higher than that of sausages stuffed in casings modified by submersion for 90 min in a solution containing 1:30 (*w/w*) soy lecithin:distilled water, 2.5% wt. soy oil, and 21 mL lactic acid per kg NaCl (17.66 ± 2.89) (*p* < 0.05). When predicting the lightness and redness of the sausage core, a partial least squares regression model developed from spectra pre-treated with a second derivative showed calibration coefficients of determination (R_c_^2^) of 0.73 and 0.76, respectively. Ten, ten, and seven wavelengths were selected as the important optimal wavelengths for lightness, redness, and yellowness, respectively. Those wavelengths provide meaningful information for developing a simple, cost-effective multispectral system to rapidly differentiate sausages based on their core colour. According to the canonical discriminant analysis, lightness possessed the highest discriminant power with which to differentiate sausages stuffed in different casings.

## 1. Introduction

Hyperspectral imaging (HSI), which can provide the spectral information along with the spatial distribution from a subject, is superior to the traditional spectroscopic methods [1]. The prediction map, as the most attractive part of HSI, enables the different attributes to be displayed from spot to spot in samples [2,3,4,5,6,7,8]. It has been intensively applied for meat adulteration detection [9,10,11,12]; monounsaturated and polyunsaturated fatty acid prediction in processed pork meats [13]; textural feature assessments of normal and white striping broiler breast meat [14]; and monitoring bacterial contaminations in chicken meat [15], the shelf-life of packaged bratwurst [16], and the pH [4], colour, [5] and triphosphate content [6] in ready-to-eat sausages. Hitherto, no study on the interior colour change due to sausages being stuffed in different modified casings has been done.

Natural casings are favoured by various sausage manufacturers due to their special cracking bite and tenderness [17,18]: the consumption of natural casings is double that of artificial casings and still dominates in the global casing market [19]. In addition to effective package [20,21,22,23,24] and cooling methods [17,25,26,27,28,29,30] to extend the shelf-lives of foodstuffs, as an efficient method for food preservation, drying is also widely employed in the food industry [31,32]. In this case, casings should be not only strong enough to hold the sausage batter, but also permeable to render water evaporation during drying [29]. Nevertheless, the high-speed and efficient sausage manufacturing is hampered by the occurrence of casing bursts, and so the properties of natural casings are requested to be improved [27]. By combining the methods used by Santos et al. [33] and Bakker et al. [34], the effects of solutions of soy oil and soy lecithin together with lactic acid in slush salt were initially investigated by Feng et al. [27]. According to the light microscopy, natural hog casings with the aforementioned treatment were observed to be more porous [27]. Previous studies addressed the microbial attributes [35,36], volatile composition [37], and physicochemical properties of sausages stuffed in modified casings [29,35,37]. HSI has showed its potential to monitor the evolution of surface sausage colour over time [2] and to determine pH, colour, and adenosine triphosphate in big Japanese sausage slices [4,5,6]. However, visualisation of the interior colour change related to hog casings with different modifications remains unexploited. Colour is an important parameter that affects the acceptance of foodstuffs by consumers to a great extent [5,38]. Conventionally, the colour is measured at a small random spot by an instrumental colourimeter. As a result, obtaining the complete colour prediction map pixel by pixel is impractical with this equipment. The distribution map generated by HSI can meet this requirement. Understanding the interior colour changes of sausages with modified casings may provide useful information on how quality changes in response to the unique modified casings.

Discriminant analysis (DA) is a method that can transform high-dimensional data into vectors as a prerequisite procedure before completing the algorithmic model [39]. It can estimate whether one of the samples belongs to defined groups according to the categories of selected variables [35,40]. According to the physicochemical and microbial attributes, sausages with different storage times and different levels of phenolic extracts from olive vegetation water were classified by DA [41]. Varrà et al. utilised the orthogonal partial last square-discriminant analysis to classify dry fermented sausages treated with ionizing radiation and non-irradiated ones, with a 100% classification rate [42]. It is thus interesting and of practical application to assess the effects of different casing modifications on the colour attribute of dried sausages using DA.

Differently from the previous research in which sausages were monitored by HSI over storage, the current study focuses on the feasibility of detecting the changes of core colour of sausages caused by different treatments (i.e., the use of different casings) using HSI. Furthermore, the relationship between colour attributes and different casing modifications is for the first time explained by discriminant analysis. The aim of current study was to establish a quantitative model relating the spectral data to the reference colour of the dried sausage core by means of partial least squares regression (PLSR). Subsequently, wavelengths with high predictive power were selected and resulting prediction maps of core colour were developed using algorithms of image processing.

## 2. Materials and Methods

### 2.1. Preparation of Samples and Measurement of Colour

Natural hog casings were purchased from a local casing company (Pakumogu.com, Niigata Prefecture, Japan) and desalted before being put into the surfactant solution composed of soy lecithin and soy oil. According to previous results on rupture force and burst pressure resistance [27], two different casing modifications were conducted. For treatment 1, the concentrations of soy lecithin (soy lecithin: distilled water) and soy oil were 1:27.5 (*w/w*) and 1.25% (*w/w*), respectively. The lactic acid concentration in solid salt was 19.5 mL/kg and the residence time was 75 min. As for treatment 2, the concentrations of soy lecithin, soy oil, and lactic acid were 1:30 (*w/w*), 2.50%, and 21 mL/kg NaCl, respectively. The residence time was 90 min. Distilled water was used to dissolve the surfactant solutions by stirring with a magnetic agitation of 325 rpm and heating at 60 °C. The hog casings were placed into the surfactant solution after it cooled to 25 °C for the corresponding reference time. Subsequently, the casings were extracted without rinsing and stored in the slush salt with lactic acid for the residence time. Before sausage production, the modified casings were rinsed for 10 min to eliminate modified solution and slush salt using distilled water.

The sausages were made with the following procedure: (1) the lean pork and back fat (Chinese supermarket, Tokyo, Japan) were cut into small pieces sterilely and mixed with seasoning extracts, Chinese white wine (55% ethanol), salt, sugar, and spice. Table 1 shows the detailed concentrations of the sausage ingredients. (2) The mixture was cured for 1 h. Afterwards, the mixture was minced once to sausage batter by using a plate (diameter: 5 mm). (3) The batter was stuffed using modified and natural desalted hog casings (as control samples) via a stuffing machine (STX-4000-TB2-PD-BL, Electric Meat Grinder and Sausage Stuffer, STX international, Tokyo, Japan). After being sectioned by twisting, the sausages were hung in an oven at 45 °C for 24 h and ageing at 20 °C in a sterilised incubator for another 24 h. The sausage sections were sterilely cut, vacuum packaged, and stored at 4 °C for 1 d.

The reference colour of the sausage core was measured by a Minolta CR-700D colourimeter (Konica Minolta Corp., Osaka, Japan) using CIE L* (lightness), a* (redness/greenness), and b* (yellowness/blueness) colour space after hyperspectral images acquirement. Before measurement, the colourimeter was calibrated by a standard white calibration plate and a standard D_65_ illuminate and 2^o^ observer. The colour measurement was conducted in triplicate.

### 2.2. Hyperspectral Imaging System

A visible near-infrared hyperspectral imaging equipment (JFE, Techno-Research Corporation, Tokyo, Japan) working in the range 380–1000 nm was utilised to line scan (push-broom) the core of the sausage. The specific system description can be found elsewhere [4,5,6]. As the white reference, a uniform, stable, and high reflectance white ceramic tile (about 99% reflectance) was used for calibrating the HSI system. As for the dark reference, calibration was performed by completely covering the camera lens with an opaque cap. A reflectance mode was used for image acquisition, with the dark room temperature controlled at 20 °C and relative humidity of 30%. The corrected reflectance (R_corrected_) was obtained according to Equation (1):

(1) $$R_{\mathrm{corrected}}=\frac{R_{raw\;}-R_{dark}}{R_{white}-R_{dark}}$$

where *R_raw_*, *R_dark_*, and *R_white_* were the reflectance images of raw, dark, and white, respectively. The sliced sausage core (diameter: 2.48 ± 0.11 cm; height: 2.10 ± 0.20 cm) was placed onto a sterilised black plastic background on a moving stage that had the speed of 2.08 mm/s. The stage was controlled by Spectrum Analyzer software (version 1.8.5, JFE, Techno-Research Corporation, Tokyo, Japan) and the spatial resolution of the acquired images was 0.75 mm per pixel. A hyperspectral image cube was produced by scanning in the direction perpendicular to the spatial plane of ImSpector spectrograph, and the hyperspectral images were processed and analysed via Spectrum Analyzer software. The illumination system, composed of a 150 W Xe lamp (Super Bright 152S, SAN-EI Electric, Osaka, Japan) and a 150 W tungsten halogen lamp (ColdSpot PCS-UHX, NPI, Tokyo, Japan), was fixed at 45° angles from the imaging area. The total spectral bands were 125 with intervals of 5 nm.

As for the region of interest (ROI), the sausage cores with different modified casings were manually selected to separate them from the background or other undesired sections. According to the ROI selection procedure described by Siripatrawan and Makino [43], the centre of each sample with a size of 50 × 50 pixels was selected, and the average spectra were used for a model development. After the multivariate statistical models were established, the images were segmented automatically: all pixels which had reflectance at 690, 685, and 685 nm for greater than 0.05 units were considered for lightness, redness, and yellowness, respectively; 75% ethanol was used for sterilising all equipment that contacted samples.

### 2.3. Model Development and Evaluation of Model Performance

One linear (partial least square regression (PLSR)) multivariate method was utilised for developing calibration models in the full spectral range of 380–1000 nm. Several spectral data pre-treatments were conducted prior to multivariate analysis (MVA) to improve the performance of the model. Those pre-treatments were standard normal variate (SNV), normalisation, multiplicative scatter correction (MSC), and first and second derivative. Two thirds of the total samples (*n* = 89) were selected for the calibration set and the remaining one third was used as the validation set.

Model performance evaluation is an important task in MVA to confirm the predictive ability and model robustness. The statistical values, namely, the mean square error of calibration (RMSEC), prediction (RMSEP), and cross validation (RMSECV), and the determination coefficients of calibration (R_c_^2^), prediction (R_p_^2^), and cross validation (R_cv_^2^) were used to estimate the predictive capabilities [9,44,45]. A model with high R^2^, low RMSE, and a small absolute difference between RMSEC and RMSECV is regarded as a good model [2]. The multivariate analyses of PLSR and all computations were completed by The Unscrambler software (X 10.4, CAMO Software Inc., Trondheim, Norway).

### 2.4. Selection of Important Optimum Wavelengths (IOW)

The weighted regression coefficients (BW) that yielded the best PLSR model were selected as the important optimum wavelengths for colour. The new calibration models were created using the selected important optimum wavelengths (those that contained large BW values irrespective of sign). The predictive ability of the model using these wavelengths was compared to the predictive ability obtained using the full spectrum. The purpose of doing this was to simplify the model and to improve its accuracy [4,5,6,45]. If the model accuracy established by important optimum wavelengths performs comparably to the accuracy obtained when using the full spectrum, then the simplified model using the IOW could be proposed for an efficient online multispectral imaging system.

### 2.5. Sausage Properties Visualisation

As aforementioned, hyperspectral imaging generates three-dimensional matrices that contain a large amount of spatial and spectral information. It is a vital approach to understand the heterogeneity of the sausage via visual appraisal in each pixel in the image. In this study, the calibration model built with the selected IOW was used to produce the distribution maps for core colour of sausages stuffed using modified and control casings. A 2D matrix was generated by unfolding the 3D HSI at the IOW. Consequently, the columns represented the selected IOW and each row stood for the spectrum of a pixel. Afterwards, the spectrum of each pixel in the HSI was multiplied with regression coefficients obtained from PLSR model. In this way, the visualisation map was constructed and can schematically display the colour heterogeneity of the sausages; it is illustrated with a linear colour scale with different colours, standing for corresponding values of predicted colour of the sausage cores. All of these visualisation procedures were computed with the MATLAB software (R2017b; MathWorks Inc., Natick, MA, USA).

### 2.6. Statistical Analysis

Sausages with different modified casings were discriminated by canonical discriminant analysis according to the colour parameters (Statistics 26, IBM, Armonk, NY, USA). One-way ANOVA was used for analysing the effects of different casing treatments on the core colour of the sausages (Statistics 26, IBM, Armonk, NY, USA).

## 3. Results and Discussion

### 3.1. Calibration Models at Full Wavelengths

As a powerful tool for multivariate data analysis, partial least square regression (PLSR) has been extensively applied in the analysis of data with collinear variables in the independent and dependent variables [4,5,6]. The correlations between spectra of the sausage samples and relevant colour parameters were established by the PLSR models. As an important parameter with which to evaluate the model performance, the range of R^2^ value between 0.66 to 0.81 has been proven to be acceptable for approximate quantitative prediction, whereas an R^2^ value between 0.82 and 0.90 is required for a good prediction. An R^2^ value over 0.91 is reported to provide excellent prediction [46]. For the lightness of core sausages, Table 2 shows that the model built with the spectra pre-treated with second derivative obtained comparably satisfactory outcomes, with R_c_^2^ of 0.73 and R_p_^2^ of 0.74, and a small absolute difference between RMSEC and RMSECV (0.05).

It was reported that the R_p_^2^ and RMSEP of the PLSR model developed to determine the lightness of cooked bratwurst pork sausages were 0.84 and 0.27, respectively [16]. The lower value of R^2^ achieved in this work may be partially attributed to the smaller image dataset (89 vs. 144) used for model training and validation. Regarding a* value, PLSR model devised by the second derivative could achieve a comparably higher R_c_^2^ result (R_c_^2^ = 0.76) with a comparably lower RMSEC (RMSEC = 1.37), whereas PLSR model developed by normalisation could reach comparably satisfactory R_c_^2^ (R_c_^2^ = 0.82) and RMSEC results (RMSEC = 1.81) for b* value. This can be due to the removal of the background noise or scattering effect reduction. Compared to a similar study, a higher R_c_^2^ value (R_c_^2^ = 0.93; RMSEC = 0.64) was also obtained when the PLSR was developed with the second derivative for predicting redness of the pre-cooked Japanese sausage [5]. Normally, pre-treatments improve the model’s performance in comparison with the model built with the raw spectra, probably because the scattering effect decreases and background noise is removed during pre-treatment procedures. For instance, the first (1st) and second (2nd) derivatives were reported to separate overlapping absorption bands [6,47], remove baseline drift and background noise [2], and eventually improve apparent spectral features [4,48]. With regard to normalisation, it is used to improve the spectral features and ensure the spectra to have an equal area under the curve, which renders the features of the spectra easy to compare in the same plot [49]. The functions of MSC and SNV are scatter correction [50]. Nolasco-Perez et al. classified ground chicken meat adulterated with pork. When using a portable NIR spectrometer, R_p_^2^ ranged between 0.01 and 0.28, and it increased to 0.77–0.84 when the classification was performed by means of NIR-HSI [51].

### 3.2. Calibration Models with IOW

The selection of representative important wavelengths is a meaningful task for simplifying the model and potentially eliminating data redundancy. The data analysis is greatly improved via this method, which facilitates the development of a simple cost-effective HSI system (such as multispectral system) or an online industrial application [9]. The reduction of the wavelengths enables one to accelerate the algorithms’ efficacies and enhance their rapid classification of the sausages according to the core colour for the industry. The statistical parameters of PLSR developed from selected IOW are shown in Table 2. Ten (385, 400, 415, 570, 690, 855, 880, 990, 995, and 1000 nm), ten (390, 400, 410, 415, 435, 515, 610, 630, 685, and 795 nm), and seven wavelengths (390, 400, 415, 420, 435, 515, and 685 nm) were selected for L*, a*, and b*, respectively.

As depicted in Table 2, the models derived from IOW had moderate performance decreases, but in some cases, possessed similar performances, which implies that the IOW were efficient enough to replace the full range of spectra for predicting of core colour of sausages with different casings. A backward feature selection was used for choosing the IOW. To be specific, if the removal of one wavelength did not significantly affect the accuracy of the developed model, then that wavelength was discarded for the development of the optimal model. In this way, the wavelengths that contained redundant information were removed and so the model was simplified: around 92%, 92%, and 94% of wavelengths were removed from the full wavelengths set for L*, a*, and b*, respectively. For the parameter of the redness, 435 and 610 nm were selected as the representative wavelengths, which is consistent with the observation of Kamruzzaman et al. [9]. The absorption band at 430 nm was reported to be related to the Soret absorption that is associated with respiratory pigment haemoglobin [52]. With regard to the absorption band at 595 nm, it related to the respiratory pigments, principally deoxymyoglobin or oxymyoglobin [53]. In comparison with a similar study on Japanese cooked sausage slices, ten wavelengths were selected for predicting redness [5]. Eight and six wavelengths were reported to predict L* and a* of pork [54], whereas a set of six wavelengths was used to predict all colour (i. e. L*, a*, and b* values) in beef, lamb, and pork [9].

### 3.3. Overview of the Spectra and Discriminant Analysis

**Figure 1:**
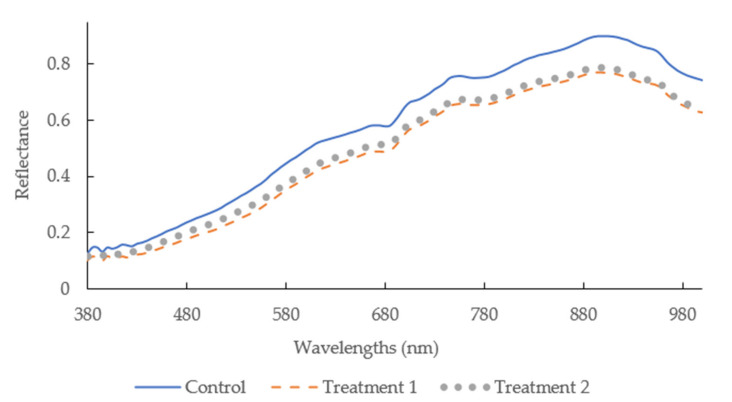
Mean spectra of the sausages with different casings in the spectral range of 380–1000 nm.

Figure 1 illustrates the mean spectra of sausages with different casings between 380 and 1000 nm. The reflectance of the sausages with treatment 1 presented the lowest reflectance, compared to that of sausages stuffed with control casing. This is consistent with the observation wherein a higher lightness for sausages with control casing (56.83 ± 5.48) was achieved. The higher water content of sausages stuffed in control casing may be attributed to this phenomenon. Due to the porous structure of modified casings, the water may evaporate via this porous structure and so concentrate the pigment, leading to less reflection. It is evident that there was a dip at 680 nm (Figure 1), which may be related to oxymyoglobin formation [2,5]. Peng and Wang stated there was a third overtone of N-H stretching between 775 and 850 nm, and there was a slope shape from 600 to 700 [55], which is related to oxymyoglobin generation [56].

As aforementioned, discriminant analysis can be utilised for evaluating whether the colour (L*, a*, and b* values) is able to discriminate the sausages with different modified casings. The relationship between colour and sausages with different casings was for the first time established. Two discriminant functions were established to separate sausages with different casings, with the correct classification of 62.90%. The two functions (Equations (2) and (3)) were as follows:

Function 1 = 0.47 [L*] + 0.14 [a*] + 0.40 [b*](2)

Function 2 = −0.62 [L*] + 0.80 [a*] + 0.32 [b*](3)

Function 1 explained 82.40% of total variance and had a higher canonical correlation (0.55) than function 2 (0.29) at a 1% significant level (Table 3), which indicates that Function 1 had a higher reliability with its higher canonical correlation. Lorenzo et al. stated that a Wilks’ lambda value was used to evaluate how well each function discriminated individuals (e.g., sausages with different casings) into groups [57]. As depicted in Table 3, function 1 possessed a low Wilks’ Lambda value (0.64) that demonstrated a pronounced discriminatory ability.

**Figure 2:**
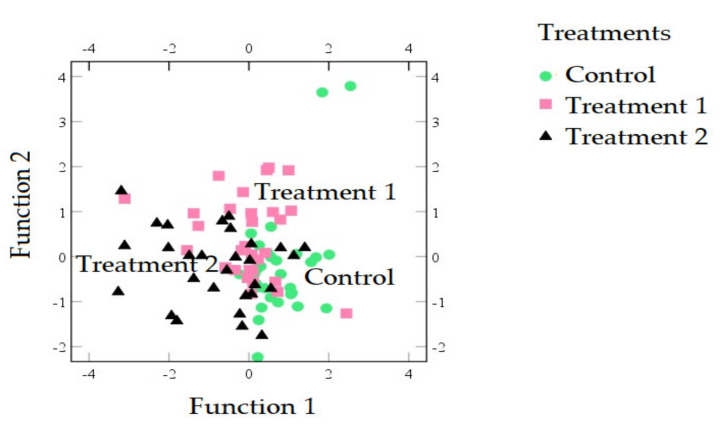
Distribution of the sausages in the coordinate system defined by the two discriminant functions used to differentiate among sausages stuffed in control (green circles), treatment 1 (pink squares), and treatment 2 (black triangles) casings.

Equations (2) and (3) also illustrate that the variable with the highest discriminant power in Equations (1) and (2) was lightness, which was followed by yellowness (*p* < 0.05). The sausages stuffed in different casings can be thus classified by lightness. This agreed with the observation wherein the lightness of treatment 1 (50.86 ± 8.90) was significantly lower than that of control (56.83 ± 5.48) (*p* < 0.05). Sausages using casings modified by treatment 2 illustrated a separation by function 2 (Equation (3)): sausages with casings modified by treatment 2 were located in the negative part of function 2, whilst modified casing (using treatment 1) sausages were in the positive part (Figure 2).

### 3.4. Visualisation of the Core Colour of Sausages

**Figure 3:**
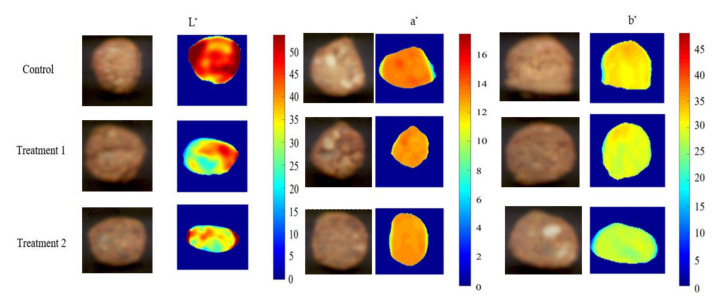
Original sausage images and a corresponding distribution map of lightness (L*), redness (a*), and yellowness (b*) changes of control casing sausages (up), and sausages stuffed in modified casings using treatment 1 (central) and treatment 2 (down) kept at 4 °C after one day storage.

The main advantage of hyperspectral imaging is its prediction map in which a pixel with a similar spectral characteristic can be displayed [58]. Combined with MVA, the distribution and concentrations of L*, a*, and b* values within the sausages stuffed in different modified casings were mapped (Figure 3). Generally, the colour of the sausages is obtained as the mean value obtained from three (or more) random spots using the conventional colourimeter for colour measurement. Due to the uneven mixture for the sausage production, the measured value may not show the entire colour of the sausage. In contrast, it is predictable that each spot is clearly displayed via prediction map [9]. According to Figure 3, the control samples presented a higher lightness value than those of sausages stuffed in treatments 1 and 2. This is consistent with the observation wherein measured lightness for control (56.83 ± 5.48) was significantly higher than that for treatment 1 (50.86 ± 8.90) and treatment 2 (51.09 ± 8.56) (*p* < 0.05). It is clear that the lightness of surrounding parts of the sausages was higher than that in the middle parts, which may have been due to the high reflection of casing. With regard to redness and yellowness, the core colour of sausages were evenly distributed.

The current study possesses potential utility for applications:

- The core colour of sausages with different modified casings were elaborated by HSI combined with MVA. The core colour changes on each pixel of casings modified by surfactant solutions were clearly displayed via distribution map. The results obtained from this technique can be used for automating the inspection and quality grading based on the core colour of the sausages by the integration of efficient image-processing algorithms in industrial machine-vision systems.
- The relationships between colour parameters and different modified casings were clearly elucidated by the canonical discriminant analysis. Although the current classification accuracy could probably be outperformed by incorporating additional features from the acquired image data or applying different data treatments, the current study provides useful information for the sausage industry. For instance, lightness possessed the highest discriminant power, followed by yellowness (*p* < 0.05). It is thus feasible to apply discriminant analysis for separating the sausages stuffed in different casings.

## 4. Conclusions

The core colour of sausages with different modified casings were characterised by hyperspectral imaging coupled with a machine learning algorithm. The canonical discriminant analysis showed lightness can separate sausages with different modified casings. The coefficient of regression of a prediction model for lightness devised by spectra pre-treated with second derivative was 0.74 with the RMSEP of 4.27. With regard to redness, the PLSR model developed by second derivative reached a higher R_c_^2^ value (R_c_^2^ = 0.76), whilst the model developed by normalisation achieved a satisfactory R_c_^2^ result (R_c_^2^ = 0.82) along with the lowest RMSEC for yellowness (RMSEC = 1.81). Ten (385, 400, 415, 570, 690, 855, 880, 990, 995, and 1000 nm), ten (390, 400, 410, 415, 435, 515, 610, 630, 685, and 795 nm), and seven (390, 400, 415, 420, 435, 515, and 685 nm) wavelengths were selected as the important optimal wavelengths for L*, a* and b*, respectively. Those representative wavelengths were used for constructing the distribution map for elaborating the core colour of the sausages with different modified casings. The lightness of sausages with control casing was significantly higher than that of sausages with modified casings (*p* < 0.05). As an emerging tool to non-destructively evaluate the core colour of the sausages with different casings, hyperspectral imaging demonstrated its powerful prediction ability and fast analysis in online and off-line inspections.

## Figures and Tables

**Table 1:** Sausage ingredients.

	Concentration (% *w/w*)
Lean pork	43.44
Back fat	20.49
Chinese white wine	26.73
Spice and seasoning	4.90
Sugars	2.50

**Table 2:** Calibration and prediction statistics for predicting core colour parameters based on full and important optimal wavelengths using PLSR with different pre-treatments.

Full wavelengths			**Calibration Group**		**Prediction Group**		**Cross Validation**	
	**Parameters**	**Pre-Treatment**	**R_c_^2^**	**RMSEC ^1^**	**R_p_^2^**	**RMSEP ^2^**	**R_cv_^2^**	**RMSECV ^3^**
	L*^6^	Raw	0.71	4.26	0.71	4.57	0.74	4.17
		Normalisation	0.72	4.21	0.73	4.38	0.75	4.04
		1st derivative	0.73	4.15	0.74	4.27	0.76	4.01
		2nd derivative	0.73	4.15	0.74	4.27	0.75	4.10
		MSC ^4^	0.71	4.26	0.71	4.57	0.72	4.31
		SNV ^5^	0.75	3.97	0.68	4.79	0.75	4.09
	a*^7^	Raw	0.59	1.46	0.55	1.51	0.59	1.44
		Normalisation	0.64	1.37	0.61	1.39	0.64	1.35
		1st derivative	0.76	1.11	0.57	1.47	0.66	1.31
		2nd derivative	0.76	1.11	0.57	1.47	0.64	1.34
		MSC	0.59	1.46	0.54	1.51	0.59	1.44
		SNV	0.73	1.19	0.58	1.45	0.69	1.26
	b*^8^	Raw	0.76	2.06	0.45	2.91	0.71	2.24
		Normalisation	0.82	1.81	0.49	2.8	0.72	2.17
		1^st^ derivative	0.65	2.52	0.44	2.92	0.68	2.35
		2nd derivative	0.65	2.52	0.44	2.92	0.72	2.19
		MSC	0.76	2.06	0.45	2.91	0.71	2.21
		SNV	0.63	2.57	0.56	2.62	0.71	2.22
Important optimal wavelengths	L*	Raw	0.66	4.63	0.70	4.62	0.69	4.50
	(385,400,415,	Normalisation	0.69	4.42	0.68	4.81	0.72	4.31
	570,690,855,880,	1st derivative	0.65	4.7	0.69	4.74	0.72	4.34
		2nd derivative	0.70	4.36	0.64	5.06	0.71	4.35
	990,995,1000)	MSC	0.65	4.73	0.66	4.95	0.71	4.38
		SNV	0.65	4.73	0.66	4.95	0.69	4.52
	a*	Raw	0.57	1.47	0.56	1.49	0.57	1.48
	(390,400,410,	Normalisation	0.61	1.42	0.62	1.38	0.61	1.40
	415,435,515,	1st derivative	0.61	1.4	0.54	1.52	0.6	1.42
		2nd derivative	0.61	1.42	0.56	1.49	0.59	1.45
	610,630,685,795)	MSC	0.58	1.47	0.63	1.36	0.59	1.44
		SNV	0.62	1.39	0.62	1.38	0.64	1.35
	b*	Raw	0.60	2.68	0.38	3.09	0.65	2.45
	(390, 400, 415,	Normalisation	0.76	2.08	0.31	3.25	0.65	2.44
	420, 435,							
	515,685)	1st derivative	0.73	2.22	0.37	3.11	0.67	2.39
		2nd derivative	0.53	2.92	0.37	3.10	0.64	2.48
		MSC	0.66	2.49	0.42	2.98	0.67	2.36
		SNV	0.66	2.49	0.42	2.98	0.67	2.36

^1^ RMSEC: the root mean square error of calibration; ^2^ RMSEP: the root mean square error of prediction; ^3^ RMSECV: the root mean square error of cross validation; ^4^ SNV: standard normal variate; ^5^ MSC: multiplicative scatter correction; ^6^ L*: lightness; ^7^ a*: redness/greenness; ^8^ b*: yellowness/blueness

**Table 3:** Colour parameters of sausages with different types of casings and main statistics of the canonical discriminant functions from colour variables.

**Colour Parameters**	**Lightness**	**Redness**	**Yellowness**	**Separation**	**Canonical Function**	**Eigenvalue**	**Variance (%)**	**Canonical Correction**	**Wilks’ Lambda**	***p* Value**
Control	56.83 ±5.48 ^a 1^	6.23 ± 2.42 ^ab^	20.26 ± 4.81 ^a^							
Treatment 1	50.86 ± 8.90 ^b^	7.09 ± 2.41 ^a^	19.75 ± 4.17 ^ab^	Treatments	Function 1	0.43	82.4	0.55	0.64	<0.01
Treatment 2	51.09 ± 8.56 ^b^	5.72 ± 1.74 ^b^	17.66 ± 2.89 ^b^		Function 2	0.09	17.6	0.29	0.92	<0.05

^1^ Averages with different superscript letters (^a^, ^b^) in the same column were significantly different (*p* < 0.05).
